# Recent setbacks in measles elimination: the importance of investing in innovations for immunizations

**DOI:** 10.11604/pamj.supp.2020.35.1.21740

**Published:** 2020-02-20

**Authors:** James L. Goodson

**Affiliations:** 1 Accelerated Disease Control and Surveillance Branch, Global Immunization Division, Centers for Disease Control and Prevention, Atlanta, Georgia, United States of America

**Keywords:** Measles elimination, immunizations, investing in innovations

## Abstract

The recent setbacks in efforts to achieve measles elimination goals are alarming. To reverse the current trends, it is imperative that the global health community urgently intensify efforts and make resource commitments to implement evidence-based elimination strategies fully, including supporting research and innovations. The Immunization Agenda 2030: A Global Strategy to Leave No One Behind (IA2030) is the new global guidance document that builds on lessons learned and progress made toward the GVAP goals, includes research and innovation as a core strategic priority, and identifies measles as a “tracer” for improving immunisation services and strengthening primary health care systems. To achieve vaccination coverage and equity targets that leave no one behind, and accelerate progress toward disease eradication and elimination goals, sustained and predictable investments are needed for the identified research and innovations priorities for the new decade.

## Editorial

The recent setbacks in efforts to achieve measles elimination goals are alarming [1]. After reaching a nadir of <100,000 estimated measles deaths globally for the first time in 2016, global measles deaths increased to > 140,000 in 2018 [1]. Since 2016, both global measles cases and incidence have steadily increased, to the highest levels since 2011. During 2016-2018, the global number of measles cases increased 167% with increases in measles incidence in five of the six World Health Organization (WHO) regions, including a 246% increase in the WHO African Region (AFR). The increase in AFR measles cases was driven by large outbreaks that occurred in Chad, the Democratic Republic of the Congo (DRC), Madagascar, and Nigeria, while other countries maintained relatively low incidence. In the AFR in 2018, coverage with the first dose of measles-containing vaccine (MCV1) was 74%, coverage with the second dose (MCV2) was 26% [2], and an estimated 52,600 children died of measles [1]. Although the AFR countries established a regional goal in 2011 to achieve measles elimination by 2020, and the World Health Assembly (WHA) endorsed the Global Vaccine Action Plan (GVAP) in 2012, with the objective to achieve measles and rubella elimination in five of the six WHO regions by 2020, it will be important to maintain political commitment and ensure substantial, sustained investments to achieve the global and regional measles elimination goals [1,3–5]. Despite overwhelming evidence of the benefits of strong immunization programs, vaccination coverage among specific populations in certain countries are stagnant or decreasing due to barriers to access, insufficient vaccine investments, and humanitarian crises [5]. To reverse the current trends, it is imperative that the global health community urgently intensify efforts and make resource commitments to implement evidence-based elimination strategies fully, including supporting research and innovations [6].

Measles and rubella elimination research priorities have been identified, including operational research and potential game-changing new tools, such as rapid diagnostic tests (RDTs) [7, 8]. Early and sustained investments in these research priorities could avoid potential future program setbacks and unnecessary excess morbidity and mortality. Evidence generated from this research and the development of effective new tools could be used to shape policy, refine strategies, and strengthen practices of the Expanded Programme on Immunization (EPI). EPI programs aim for control and elimination of vaccine-preventable diseases and reduction of morbidity and mortality [1, 9–11]; elimination efforts reinforce a data-driven focus to reach vaccination coverage and equity targets. When fully resourced, EPI and related research can readily identify gaps in immunization services based on data and field experience and drive innovation through an iterative process of developing and implementing new strategies, field testing, analyzing data, and making evidence-based program adjustments. Strategic recommendations based on the evidence are endorsed by policy-setting bodies including the global WHO Strategic Advisory Group of Experts (SAGE) on Immunization, and regional and national Immunization Technical Advisory Groups.

### Strengthening immunization service delivery

Measles outbreak investigations, case-based surveillance data analysis, vaccination coverage surveys, systematic EPI reviews, vaccine-preventable disease impact assessments, and cost-effectiveness studies provide opportunities for research to generate evidence for refinement of elimination strategies. The published literature is rich with evidence that supports simultaneous EPI strengthening and measles elimination, including the impact of the recently updated Reaching Every District strategy; integration of other public health interventions with immunizations service delivery including supplemental immunization activities (SIAs) [12–14]; incorporation of mobile phone use, electronic immunization registries, and recall and reminder systems for vaccination messaging [15]; novel approaches to reduce vaccination dropouts and missed opportunities for vaccination (MOVs); establishing a second-year-of-life (2YL) platform; and SIA microplanning to reach un- and under-vaccinated children [16].

In 2009, an accumulation of evidence led to the WHO recommendation that all countries provide two doses of measles-containing vaccine [17]. Globally, estimated MCV2 coverage increased from 18% in 2000 to 69% in 2018, largely because of an increase in the number of countries providing MCV2 from 98 (51%) in 2000 to 171 (88%) in 2018 [1]. In many AFR countries, MCV2 introduction was the first routine EPI vaccine given to children beyond infancy that required establishing a 2YL clinic visit for scheduled vaccination [2, 18]. Multiple post-introduction evaluations for MCV2 and 2YL initiatives have led to an accumulation of information that can be used to strengthen EPI operations, including using the MCV2 vaccination visit to catch up on previously missed doses of all vaccines [19–22]. Providing two doses of measles-containing vaccine (MCV) to all children has also further highlighted the advantages of using 5-dose vials rather than 10-dose vials of MCV. In 2019, an important comprehensive study by John Snow, Inc. (JSI) showed that using 5-dose vials compared with 10-dose vials was associated with a substantial increase in MCV2 coverage, a significant decrease in MCV1-MCV2 dropouts, and a significantly lower MCV wastage rate (16% compared with 30%). Furthermore, the wastage-adjusted vaccine price per dose was $0.98 for 5-dose vials compared with $0.94 for 10-dose vials, and there was only a 4.9% increase in cold chain space requirements for using 5-dose vials [23]. In November 2019, after careful review of evidence, including the JSI study, the African Regional Immunization Technical Advisory Group now encourages the use of 5-dose vials of MCV in appropriate settings [24].

### Risk mitigation and preventive actions

Advances in serological surveys, disease mathematical modeling, measles-susceptibility profiles, and measles risk assessments have facilitated identifying measles population immunity gaps and sub-national areas at-risk [25–30]. However, the results of these studies could be better used to support timely preventive actions, including SIAs to mitigate risk before large measles outbreaks occur. For example, the prescient results from analysis of data from serological surveys published by Winter et al. indicated the risk for a massive measles outbreak in Madagascar; in hindsight, it could have led to immediate preventive action or a timelier outbreak response [27]. Similarly, given WHO and United Nations Children’s Fund (UNICEF) estimates of national immunization coverage indicating low population immunity in DRC, a decision could have been made to repeat a measles SIA earlier than the three-year interval between the 2016 and the 2019 measles SIAs, at least mitigating the scale of the current outbreak.

Periodic nationwide SIAs are a long-established cornerstone of elimination efforts that include special strategies and microplanning for reaching zero-dose and under-vaccinated children previously missed by routine immunization services. Starting in 2016, however, global measles donor funds were redirected toward organizations that focused on health systems strengthening rather than measles elimination [31]; this was followed by funding reviews that suggested that countries downsize nationwide measles SIAs to subnational SIAs, extend the interval between SIAs, or restrict SIA target age groups to young children [32]. It was thought that the cost savings from the proposed smaller SIAs could then be used flexibly on additional immunizations systems strengthening activities in districts not included in the SIA [33]. However, pilot testing of this approach found that data quality was not high enough to support decisions to exclude certain districts from SIAs.

SIA frequency and target age groups should be based on epidemiological analyses, and adequate resources made available to ensure optimal implementation of the indicated target population and SIA timing [34, 35]. Previous published studies in the AFR have shown negative impacts of narrow target age groups, delayed SIA implementation, subnational phased implementation, and long gaps in SIA frequency [36–40]. The impact of suboptimal SIA implementation can be devastating, including, for example, the deadly measles epidemics that have continued to occur predictably in DRC, including 327,959 reported cases and 6,256 reported deaths during December 31, 2018-January 19, 2020 [41]. Any proposed alternative strategies, including methods that aim to identify subnational target populations, limit the geographic scope, or decrease the frequency of SIAs should be carefully evaluated to provide evidence of impact on disease burden and long-term cost effectiveness compared with existing elimination strategies.

### Changing measles epidemiology, vaccine effectiveness and immunity

Measles epidemiology has changed over time, following decreases in measles incidence in all regions since 2000. Studies have documented this changing epidemiology, including in the AFR [42], and recent reviews have described some fundamental aspects of current measles epidemiology related to elimination strategies [43–45]. For example, with increased vaccination coverage, there has been a shift from protective immunity developing primarily after wild-type measles virus infection to one that is derived from vaccination, with less opportunity for natural boosting from exposure to wild-type measles virus. This has resulted in a shift in measles-susceptibility to older age groups, including young adults [8, 38, 42]. In addition, infants become susceptible to measles at an earlier age [46, 47]. Studies have shown that maternally derived measles antibodies passively transferred to infants via the placenta from vaccinated mothers are lower and wane faster below the protective threshold than from mothers who had measles from wild-type infection [45, 46, 48]. A recent study in an elimination setting found 92% of infants became susceptible to measles by 3 months of age [46].

Similarly, a recent review of the measles reproduction number (R0), the measure of transmissibility that drives herd immunity and subsequent vaccination coverage levels needed to interrupt measles virus transmission, showed that R0 estimates vary considerably by setting and more widely than the often-cited 12-18 range, and they are dependent on context-specific factors including population density, birth rates, and age-mixing patterns [49]. Better understanding of the contributors to transmissibility in various settings may improve elimination efforts in specific contexts.

With changing measles-susceptibility, a recent review of the effect of age at first dose and time since vaccination on measles vaccine effectiveness (VE) was completed. It showed that, in measles-endemic settings, one-dose VE increased by 1.5% (95% confidence interval=0.5, 2.5) for every month increase in age at first dose and found no evidence of waning VE. More data, however, are needed to answer the question of whether measles VE wanes in measles-elimination settings [50]. Recent studies in some elimination settings have suggested that waning immunity among older children and adults might have led to emerging measles susceptibility and that breakthrough infections might have played a role in some outbreaks. However, this phenomenon has been observed only in a small number of elimination settings that likely experienced gaps in cold chain and/or vaccine mishandling in the past [51–53]. Detailed case investigations and laboratory evaluations are needed to confirm measles cases as breakthrough cases and provide clearer evidence of potential waning measles immunity, to support decisions to revaccinate populations experiencing re-emerging measles susceptibility [54, 55].

Measles virus infection leads to severe viremia and lymphopenia and can cause immunosuppression that can last for months to years [43]; however, the long-term impact of measles on the immune system is not fully understood [56]. Recent studies have demonstrated that measles virus can infect up to 70% of memory T-cells during the first 3-10 days after infection [57, 58], and measles virus infection diminishes specific preexisting antibodies that were providing protection from other pathogens [51–60]. Further studies are needed to quantify the impact and implications of the long-term susceptibility to other pathogens caused by measles infection.

### Potential game-changing tools

Important innovative tools are on the horizon, including a measles rapid diagnostic test (RDT) and a measles-rubella (MR) vaccine microneedle patch that are among the highest priorities for measles and rubella elimination research [8]. A measles RDT is currently being field tested in several studies in Ghana, India, Malaysia, and Uganda, and a rubella RDT is in development. RDTs have the potential to substantially reduce time to case confirmation and fundamentally change approaches to outbreak response and infection control measures [61]. For example, rapid confirmation of a suspect measles outbreak by a district health officer or diagnostic testing of suspect measles cases at the clinic could lead to more timely outbreak response immunization, and appropriate triaging and isolation of cases in hospitals and health centers. The MR microneedle patch is widely recognized as a potential game-changer for elimination strategies. The MR patches will require minimal storage and disposal capacity, are easily transported, do not require reconstitution with diluent, cannot be re-used because they dissolve in the skin, do not generate sharps waste, and are easily administered, permitting vaccination by minimally trained personnel [62]. The patch will eliminate adverse events following immunizations due to human error during reconstitution and make house-to-house vaccination campaigns possible, a key strategy for elimination and eradication efforts [63, 64]. Despite the clear potential positive impact on vaccination coverage and equity, and long-standing urgent calls for investments in MR microneedle patches [65, 66], securing sustained predictable funding commitments has been challenging, adding unnecessary years to licensure and use [67]. The current optimistic timeline for developing and commercializing MR patches, even with timely funding, is estimated to be 7-8 years. Novel product development to improve upon existing products often requires formation of global public-private partnerships, similar to the partnership that supported development of the N. meningitides group A vaccine, MenAfriVac™, to firmly establish the public health need, advocacy, and to make the business case for shared costs and risks of the development process [68].

### Build synergy for common goals

With the decade of vaccines coming to an end in 2020, global immunization partners are establishing the “Immunization Agenda 2030: A Global Strategy to Leave No One Behind” (IA2030) [69] to be approved by the WHA in May 2020 for the next decade. This new global guidance document builds on lessons learned and progress made toward the GVAP goals. The IA2030 includes research and innovation as a core strategic priority and identifies measles as a “tracer” for improving immunisation services and strengthening primary health care systems. Measles has proven to be an effective tracer for EPI performance and as a driver for efforts to strengthen health systems and innovations [70]. Key factors that make this possible include: 1) very high measles vaccine effectiveness, 2) very high transmissibility of measles virus among unimmunized people, and 3) the absence of silent measles virus transmission, a characteristic which distinguishes measles from polio. All measles cases have a well-defined clinical presentation of maculopapular rash and fever, sometimes seen with the pathognomonic Koplik spots; therefore, are detectable by disease surveillance. Measles epidemiology accurately reflects measles susceptibility in the population, thereby identifying areas and communities with low vaccination coverage. Also, measles is frequently the first vaccine-preventable disease detected when weaknesses in immunization service delivery occur. Therefore, measles is often referred to as the “canary in the coalmine” for EPI and as such, can be effectively used as a signal and driver for overall immunizations systems strengthening [71]. Achieving measles elimination in AFR will focus efforts to deliver two doses of measles vaccine safely and effectively to ≥95% of children in a timely manner, as well as detect, prevent, and respond effectively to measles cases and outbreaks. These efforts can dovetail synergistically with the aims of the Global Health Security (GHS) and the Universal Health Coverage (UHC) agendas to strengthen primary health care systems, immunizations and preventive services, disease surveillance, and outbreak preparedness and response capacity [3, 72–75]. To achieve these common goals, attain vaccination coverage and equity targets that leave no one behind, and accelerate progress toward disease eradication and elimination goals, sustained investments are needed for the identified research and innovations priorities.

## Competing interests

The author declares no competing interests.
